# How Can We Better Evaluate Complex Global Health Initiatives? Reflections From the January 2014 Institute of Medicine Workshop

**DOI:** 10.9745/GHSP-D-14-00184

**Published:** 2015-05-20

**Authors:** Sangeeta Mookherji, Kate Meck

**Affiliations:** ^a^​ George Washington University, Department of Global Health, Washington, DC, USA; ^b^​ Institute of Medicine of the National Academies, Washington, DC, USA

## Abstract

An IOM workshop on evaluation design drew on recent evaluations of 4 complex initiatives (PEPFAR; the Global Fund to Fight AIDS, TB and Malaria; the President's Malaria Initiative; and the Affordable Medicines Facility-malaria). Key components for good evaluations: (1) a robust theory of change to understand how and why programs should work; (2) use of multiple analytic methods; and (3) triangulation of evidence to validate and deepen understanding of results as well as synthesis of findings to identify lessons for scale-up or broader application.

The context for global health interventions and their evaluations has become more complex in the 21st century. Donor assistance for global health has increased dramatically in the last 15 years, and most of these resources are channeled through complex global health initiatives that target various health outcomes through a multitude of interventions, implemented by diverse partners in multiple countries and regions of the world. Rigorous evaluations are needed to assess the achievements of these initiatives and to justify and increase investments in them. Large-scale evaluations of complex global health initiatives are relatively new, and knowledge of how to improve such evaluations is needed.

Our recent experiences have repeatedly exposed the challenges in evaluating global health initiatives that involve any degree of complexity. Health initiatives are often implemented at national scale, and reasonable comparison groups cannot be identified. Even though many initiatives monitor progress toward outputs and outcomes using performance- or results-based strategies, these strategies rarely provide insight as to whether or how different implementing partners were able to achieve success; what problems were or were not addressed successfully; and how situational variability affected successes and challenges. Often, we simply don’t gain the learning needed from evaluations about how a complex intervention worked or did not, and how implementation context affected intervention success.

We need to do better with designing and conducting more complex evaluations of complex global health initiatives and to do this in creative yet robust ways that allow us to understand both the complexity and the specificity of implementation context. This will require support and input from program implementers, evaluators, and commissioners of evaluations. In this article, we report on key messages from a workshop on “Evaluation Design for Complex Global Health Initiatives,” convened by the Institute of Medicine (IOM) in January 2014, during which workshop participants discussed just how this might be done.

Evaluations of complex global health initiatives need to provide information on both the complexity and specificity of implementation context.

## OVERVIEW OF IOM WORKSHOP

What information do we need to scale-up and sustain a success story? What can failure to achieve expected health effects teach us about how implementation conditions and the political landscape contributed to the observed results? What can evaluators do better when evaluating complexity in global health initiatives?

The IOM asked workshop participants to address these important questions. The goal of the workshop was to extend evaluation methodology by capturing lessons learned from recent large-scale, complex, multinational global health initiatives. The workshop derived lessons learned from the execution of evaluations and discussed how to apply lessons to future evaluations.^1^

Four recent evaluations served as core examples for the workshop:

- The US President’s Emergency Plan for AIDS Relief (PEPFAR)^2^
- The Global Fund to Fight AIDS, Tuberculosis and Malaria (Global Fund)^3^
- The US President’s Malaria Initiative (PMI)^4^
- The Affordable Medicines Facility-malaria (AMFm)^5^

For the core examples, “large-scale” was defined as having total cumulative budgets, over multiple years, in at least the hundreds of millions of US dollars. “Multinational” meant implementation on a global scale, including multiple countries and regions or subregions of the world. “Complex” referred to several dimensions of the initiative: multiple activity components; varied settings for implementation of different sets of activities; systems-strengthening efforts; capacity building; efforts to influence policy changes; use of health diplomacy to achieve the aims of the initiative; and implementation at multiple levels through a large number of diverse, multisectoral partners at the country level.^1^

The IOM invited representatives from each of the 4 core evaluations to elucidate the decision making process and the options that were available to develop and implement a credible and rigorous evaluation that was also feasible, affordable, and maximally matched to the priority evaluation questions, aims, and audiences. In addition, representatives from other evaluations that met some, but not necessarily all, of the criteria above (Box), were asked to serve as panelists and to present their experiences and perspectives on the methodological challenges they addressed. Evaluation experts from other relevant disciplines, including education, climate and environment, and other non-health areas, along with commissioners of evaluations, held honest discussions over the course of 2 days regarding the challenges of and lessons learned from conducting evaluations of complex, large-scale, multinational global health initiatives.

**BOX. Global Health Initiatives and Evaluations Represented at IOM Workshop, January 2014**

Four recent evaluations of large-scale, multinational, complex global health initiatives served as core examples for the workshop:

- Global Fund to Fight AIDS, Tuberculosis and Malaria (Global Fund)
- US President's Malaria Initiative (PMI)
- Affordable Medicines Facility–malaria (AMFm)
- US President's Emergency Plan for AIDS Relief (PEPFAR)

Other evaluation experiences were presented that had addressed issues of complexity and scale:

- Global Environment Fund (GEF)
- Gavi (formerly, the Global Alliance for Vaccines and Immunisation)
- Integrated Management of Childhood Illness (IMCI)
- Accelerated Child Survival Development (ACSD) Program
- Africa Routine Immunization System Essentials (ARISE)
- Saving Mothers, Giving Life
- Avahan in India
- Expanded Quality Management Using Information Power (EQUIP)

The IOM workshop proceedings were published in June 2014.^1^ We used the published proceedings as a data source and applied 34 codes using NVivo to distill the large amount of information in the proceedings text into key messages. (See the supplementary materials for details about the coding methodology and summary.) Although the proceedings are comprehensive, the publication organizes the workshop information chronologically. The free-form discussion sessions and information sharing, as well as the linkages among dedicated panel sessions, meant that, for example, relevant lessons on addressing context could be found throughout the 115-page text. We used the coding exercise to further organize the information in the proceedings document to make the important lessons and messages more accessible for practitioners of global health evaluations. Both authors were workshop participants, and one a panelist, so we also used our own participant observations to triangulate and synthesize the following lessons and recommendations from the workshop.

## KEY MESSAGES ABOUT EVALUATING COMPLEX GLOBAL HEALTH INITIATIVES FROM THE IOM WORKSHOP

Three areas of focus to improve future evaluation of complex global health initiatives were identified at the IOM workshop: (1) the importance of theory of change for grounding complex evaluations; (2) the need to use multiple methods to address complexity; and (3) the need to focus more on triangulation and synthesis of findings.

### Theory of Change Grounds Complex Evaluations

Workshop participants confirmed the critical role of theory of change (ToC) that depicts the series of expected causal steps between activities and impacts for optimizing complex evaluations. Participants used various terms—logic models, results chain, causal chain pathway, program impact pathway, program theory, and program impact theory—but whatever the name, workshop participants confirmed that a ToC is most useful when it identifies the links in program planning, implementation, and delivery and especially the central assumptions, implementation conditions, and contextual factors that are likely to influence a complex initiative. The panel discussions confirmed the crucial need for developing a ToC for all program purposes: design, implementation, and continuous performance improvement, as well as for evaluation.

A theory of change depicts the series of expected causal steps leading from program activities to outcomes and impacts.

A critical early step in the PEPFAR evaluation was developing a ToC (program impact pathway) that incorporated the various inputs into the PEPFAR initiative, including the considerable financial and technical assistance resources and the strategies, guidance, and planning activities that support implementation of the initiative.^2^ This ToC was then simplified to cover the diversity of programs and used to communicate to a variety of audiences about the evaluation. The ToC helped the evaluation committee explore the feasibility of the various designs and methods that might be used in the evaluation, and then it was used during analysis to help the committee understand the impacts of PEPFAR in terms of proximal, intermediate, and distal effects.^1^

The AMFm evaluation team developed a ToC to depict the causal pathways through which AMFm interventions were intended to work. This ToC was used to target the collection of quantitative and qualitative data that would be used to prepare case studies for each country, thereby providing a standard framework for evaluation across countries for data collection and later for analysis. The evaluation team collected qualitative data through interviews with key stakeholders from the public and private sector and a review of key documents to understand the AMFm implementation processes and contextual factors identified by the ToC within each country. The evaluation team used quantitative data from the outlet surveys on process-related outcomes, such as coverage of training and exposure to communications messages. These were analyzed separately for each country case study and then synthesized into findings across countries.

ToCs helped evaluators address the critical role of context. The Global Fund explained that it takes an open approach to causation that considers alternative hypotheses involving context, often starting with impact and outcomes and working back along the causal chain pathway to identify other change factors that could be dependent on context.^1^ In the PEPFAR evaluation, the issue of context surfaced early during design of the evaluation, as the committee understood that the program impact pathway for PEPFAR operations was embedded in the context of many other factors in each country; the evaluation team then examined a variety of indicators across countries to give a contextual background to PEPFAR’s operating environment. Contextual issues in countries visited were explored through the significant qualitative data collection component of the PEPFAR evaluation.^1^ One of the lessons from the AMFm evaluation was the importance of documenting the process of implementation using a ToC model when large-scale, complex interventions are being implemented in a messy, real-world setting.^1^ In fact, the AMFm evaluation found that context probably made the most crucial difference between countries in terms of performance.

Theories of change help evaluators address the critical role of context.

Workshop participants continually emphasized the need to pay more attention to context and challenged each other to “really unpack what the notion of context means,” including recognizing that contextual issues arise at micro-, meso-, and macro- levels and that they can evolve over time. The participants called for better differentiation between contextual “constants,” which cannot be influenced; contextual factors that can be influenced; and contextual factors that directly support the observed changes. If evaluators parse context in this way, it may become clear that controlling for contextual complexity through study design may not be desirable in many evaluation situations, because this could strip out the very things that are important mechanisms for change.^1^ Including contextual factors and categorizing them this way in a comprehensive ToC is the crucial step that allows comprehension of how and why a program worked the way it did, and whether it would work that way somewhere else or at larger scale.

### Using Multiple Methods Helps Address Complexity

Workshop participants affirmed that for complex global health initiatives, evaluations need to use a methodological approach that includes multiple data collection and analysis methods, often through nested study designs that combine qualitative and quantitative approaches to data collection and analysis, to address a variety of evaluation questions. “Multiple” and “mixed” methods described how the 4 large-scale evaluation examples used a number of complementary methods to arrive at evaluation findings and conclusions. Most panelists agreed that 2 sources of data are often not enough to have confidence in the results from a complex evaluation—hence, panelists mostly referred to “multiple” methods, and not just mixed methods.^1^

Recent evaluations of 4 complex global health initiatives used multiple, complementary research methods.

The critical question faced by each of the 4 core examples was how to ensure that these multiple methods were mixed, and not mixed up or parallel. These issues had to be addressed during both the design and the analysis stages, to ensure that any qualitative and quantitative data collection conducted in parallel were linked during analysis and that data from different sources were collected in a way to allow triangulation later on.

The end result of the PEPFAR design phase was a “hybrid” evaluation that included retrospective and cross-sectional elements, as well as time series and time trend data and nested in-depth approaches on different topical areas. The approach used multiple sources of qualitative and quantitative data to balance the limitations of each other.^1^ One of the primary challenges was that few data sources were available consistently across the entire PEPFAR program. The PEPFAR evaluation team instead focused on using the best methods for each type of data and matched the appropriate analytical methods to different types of data.

The AMFm team learned the importance of standardizing data collection and analysis methods to assure quality. The team also recognized the challenges of mounting a large primary data collection exercise that is constrained on the one hand by epidemiology and logistics and on the other hand by dependency on countries for data that may not be forthcoming within the required timeline. Finally, relying on secondary analysis for key outcomes was a limitation but one that could not be overcome because of budgetary and time restrictions.

The PMI evaluation started with a qualitative management review exercise in which the primary sources of data were key stakeholder interviews with PMI leadership, as well as global, regional, and in-country stakeholders who benefit from the initiative. While this was a relatively straightforward activity, the evaluation team soon recognized that it needed to explore other data to understand what the program was actually doing. This included both quantitative data about key interventions and qualitative data about strengthening health systems and capacity building within national malaria control programs; for the latter, it was useful to look at program data from other donors supporting malaria control, such as the Global Fund, as yet another source of data. The Global Fund data, for example, might show the total number of drugs and nets purchased and delivered to a country, but to understand distribution and consumption, the PMI evaluation team had to look to other sources. It was more difficult to understand whether PMI was strengthening health systems or national malaria control efforts. The evaluation team considered the methodological trade-offs when deciding on the right mix of qualitative and quantitative methods and use of routine program monitoring data, and the need to balance these trade-offs to generate results that were useful and informative.

To implement multiple methods well and produce useful evaluations of complex interventions, multidisciplinary teams of evaluators are needed. Evaluators and commissioners of evaluations noted it is difficult to assemble teams comprising individuals with the wide range of skills required for evaluating complex programs. Working with a multidisciplinary team of investigators to do good multidisciplinary science also is a major challenge. The approach taken by the PMI evaluation team was to match the team member with the dominant relevant expertise for each key issue with someone who had a completely different set of expertise—someone with a different perspective. The result was a richer understanding of the analysis. Investing in multidisciplinary collaboration and capacity building for complex evaluation, especially among local partners, often required more time and focus than the evaluation study time frame and budget allowed; rectifying this imbalance will require evaluation commissioners to develop more realistic budgets and timelines, preferably done in partnership with evaluators.

Multidisciplinary teams of evaluators are needed to implement the multiple research methods needed for large-scale evaluations.

### Triangulation and Synthesis Validates and Builds Confidence in Evaluation Findings

Using multiple methods to address complexity demands triangulation. There is no other way to consolidate multiple sources of qualitative and quantitative information, collected and analyzed using different methods, and to arrive at useable evaluation findings and conclusions. In the evaluation process, when data are unreliable, scarce, or inconsistent across implementation settings, methodological triangulation is necessary to accurately interpret data from different sources and methods. Workshop participants distinguished between *triangulation*, which is the analytical process to validate results from different sources of data or address discordance in findings related to different sub-questions of an evaluation, and *synthesis*, which is the analytical process that pulls together findings across different units of analysis to identify context-neutral findings that can be used in other settings or for scale-up.

Triangulation validates results from different sources of data while synthesis brings together findings from different units of analysis.

All 4 of the large-scale evaluations used purposive, case-based selection of study units, either exclusively or as part of their multiple methods approach. Findings from these nested studies did not use statistical methods but had to be integrated with findings from other nested studies that did, in a robust way to support generalizability of the overall findings. This could only be done through a systematic triangulation process and synthesis of context-specific and context-neutral findings.

In the core workshop examples, triangulation was done to validate the results and deepen and broaden the understanding and insights gained from the evaluation findings. All the triangulation and synthesis approaches described in the core examples were grounded in the evaluation’s purpose and ToC. The PEPFAR evaluation team conceptualized data triangulation happening at the levels of analyses and interpretation, instead of data collection, since data across the entire PEPFAR program were scarce. They were careful to document what analytical methods were used for what data, to ensure that the analysis of data from the multiple methodological sources was as transparent, purposeful, and rigorous as possible. The PEPFAR evaluation team noted that the quality and rigor of the causal contribution analysis were improved by using triangulation for the different types of data and different analyses. When combined with the ToC, this provided a solid basis to help determine not just whether PEPFAR was affecting health outcomes but also how and why.

The AMFm team distinguished that triangulating data from multiple sources deepened the evaluators’ understanding of within-country results, while synthesizing the findings across countries contributed to an understanding of how an AMFm intervention could work in other countries in the future, by identifying the key factors that contributed to strong performance and those that were associated with weaker performance.

Evaluations from the Global Environment Fund (GEF) examined progress from outcomes to impact, usually in the face of sparse data, in particular country program objectives and indicators and national statistics on environmental indicators and data series over the 20 or so years of a typical evaluation time frame. To overcome this data scarcity, GEF uses a standard set of data-gathering methods and tools that include desk and literature reviews, portfolio analyses, and in-depth interviews, in addition to GEF-specific methods, such as analyses of a country’s environmental legal framework. All these methods are deployed within the context of an evaluation matrix that the GEF develops for each evaluation, which then feeds into a triangulation matrix.^1^ In the triangulation process, the evaluation team brainstorms question-by-question to populate the matrix and discuss which findings are credible and which need further analysis. After the brainstorming meeting, the team tries to confirm or challenge the key preliminary evaluation findings and identify what else can be done to fill in the missing information, using the GEF’s theory-based approach to examine progress from outcomes to impact.

Presenters of the 4 core examples emphasized the importance of the ToC in supporting robust triangulation and synthesis processes. For example, in reaching the conclusion that context was most likely the most important contributing factor to performance differences across countries, the AMFm evaluation relied on the ToC to help them parse the findings. The PEPFAR evaluation was able to determine not only whether PEPFAR was affecting health outcomes but also how and why, through a process of triangulation of multiple sources of data and multiple analyses, in combination with the ToC.^1^ The PEPFAR ToC was the key to high-quality, rigorous causal chain analysis that provided a solid evidence base for the evaluation’s conclusions and recommendations.

A theory of change supports robust triangulation and synthesis processes.

Workshop participants expressed that evaluators need better guidance on triangulation and synthesis and that implementers and commissioners need to understand what is involved with these analytical processes, because they take time, yet are crucial for producing the evaluation results that are expected. Systematic triangulation and synthesis across study units is important for increasing confidence in the reliability, credibility, and applicability of evaluation findings, especially when case-based, non-probability–based evaluation designs are used. However, participants agreed that evaluators should continually assess the purpose of the triangulation or synthesis exercise: Is it enriching survey data with qualitative findings? Assessing hypotheses emerging from analysis of one data source with results from another? Explaining unexpected results using another source of data? Verifying or rejecting conclusions based on the concordance or discordance of results from different sources of data? Each requires a different process of triangulation and synthesis, and each requires sufficient time and input from the multidisciplinary team that must be adequately budgeted and planned by evaluators and evaluation commissioners.

Systematic triangulation and synthesis is important for increasing confidence in the reliability, credibility, and applicability of evaluation findings.

## WHY ARE THE KEY MESSAGES FROM THE IOM WORKSHOP IMPORTANT?

Each of the 3 areas identified at the IOM workshop presents an important opportunity for evaluators, commissioners, and implementers to do better with evaluating large-scale and complex global health initiatives. Addressing the 3 critical areas identified here will become increasingly important in the future: our evaluation questions, the settings in which we implement, and the initiatives themselves, are becoming more complex and interlinked. Evaluators, program implementers, and commissioners of evaluations must address the issues identified through the IOM workshop, and soon. We need evaluations of large-scale global health initiatives that speak to both their complexity and the specificity of implementation context in order for the findings to be useful. We need to improve how we commission, conduct, consume, and convert evaluation findings from complex global health initiatives to improvements in implementation and impact. Without sufficient and immediate attention to these 3 areas from all parties, we risk continued low returns on our evaluation investments and minimal progress in building the evidence base for improved global health.

## Notes

**Peer Reviewed**
